# Fine Mapping of the Wheat Leaf Rust Resistance Gene *Lr42*

**DOI:** 10.3390/ijms20102445

**Published:** 2019-05-17

**Authors:** Harsimardeep S. Gill, Chunxin Li, Jagdeep S. Sidhu, Wenxuan Liu, Duane Wilson, Guihua Bai, Bikram S. Gill, Sunish K. Sehgal

**Affiliations:** 1 Department of Agronomy, Horticulture & Plant Science, South Dakota State University, Brookings, SD 57006, USA; harsimardeep.gill@sdstate.edu (H.S.G.); jagdeeproots@gmail.com (J.S.S.); 2 National Engineering Laboratory of Wheat, Wheat Research Institute, Henan Academy of Agricultural Sciences, Zhengzhou 450002, China; licx82@yahoo.com; 3 The State Key Laboratory of Wheat and Maize Crop Science, College of Life Sciences, Henan Agricultural University, Zhengzhou 450002, China; wxliu2003@gmail.com; 4 Wheat Genetics Resource Center, Department of Plant Pathology, Kansas State University, Manhattan, KS 66506, USA; dlwil@ksu.edu (D.W.); bsgill@ksu.edu (B.S.G.); 5 USDA-ARS, Hard Winter Wheat Genetic Research Unit, Manhattan, KS 66506, USA; guihua.bai@ars.usda.gov

**Keywords:** wheat, *Aegilops tauschii*, *Lr42*, disease resistance, molecular mapping, KASP markers, marker-assisted selection

## Abstract

Leaf rust caused by *Puccinia triticina* Eriks is one of the most problematic diseases of wheat throughout the world. The gene *Lr42* confers effective resistance against leaf rust at both seedling and adult plant stages. Previous studies had reported *Lr42* to be both recessive and dominant in hexaploid wheat; however, in diploid *Aegilops tauschii* (TA2450), we found *Lr42* to be dominant by studying segregation in two independent F_2_ and their F_2:3_ populations. We further fine-mapped *Lr42* in hexaploid wheat using a KS93U50/Morocco F_5_ recombinant inbred line (RIL) population to a 3.7 cM genetic interval flanked by markers *TC387992* and *WMC432*. The 3.7 cM *Lr42* region physically corresponds to a 3.16 Mb genomic region on chromosome 1DS based on the Chinese Spring reference genome (RefSeq v.1.1) and a 3.5 Mb genomic interval on chromosome 1 in the *Ae. tauschii* reference genome. This region includes nine nucleotide-binding domain leucine-rich repeat (NLR) genes in wheat and seven in *Ae. tauschii*, respectively, and these are the likely candidates for *Lr42*. Furthermore, we developed two kompetitive allele-specific polymorphism (KASP) markers (*SNP113325* and *TC387992*) flanking *Lr42* to facilitate marker-assisted selection for rust resistance in wheat breeding programs.

## 1. Introduction

Wheat is one of the leading staple foods worldwide, providing one-fifth of the calories and protein to more than 4.5 billion people [1]. Wheat production is constrained not only due to changing climate, but to a great extent by the emergence of new and more virulent races of economically important pathogens. Several diseases and insect pests, including leaf rust (caused by *Puccinia triticina* Eriks), threaten sustainable wheat production in the major wheat-growing areas of the world [2]. In Kansas alone, the leaf rust epidemic of 2007 caused yield losses of 13.9% in winter wheat [3]. Yield losses are attributed to fewer kernels, aggregated by lower kernel weight [2], and losses can be severe if wheat is infected early in development and may reach epidemic proportions in susceptible cultivars under favorable conditions [4]. Diving into history, one can find a reference to leaf rust in the Bible and literature of classical Greece and Rome [5]. Also, the existence of prevalence of this disease from that era until today indicates that this pathogen has evolved along with wheat or other grass species and there has been no permanent solution to control this disease, and likewise for other rusts.

Breeding for rust resistance is considered as one of the most economical approaches to manage rust diseases, and wheat breeding programs throughout the world are deploying rust resistance genes in commercial cultivars. The populations of *P. triticina* are reported to be highly variable in North America [6], with many different virulence pathotypes or races detected annually. Therefore, a more durable approach for long-lasting resistance is the pyramiding of different race-specific and race non-specific genes in single cultivars [7]. However, combining different resistance genes using phenotypic selection is a challenging and cumbersome process. Molecular markers can be very useful for simultaneous stacking of different resistance genes. Thus, the availability of tightly linked molecular markers for different genes is essential for facilitating the stacking of these genes into a single genetic background.

To date, around 80 leaf rust resistance (*Lr*) genes have been formally reported in wheat and its wild relatives [8]. *Aegilops tauschii* Coss, the D-genome donor of wheat, has been a rich source of resistance genes [9,10] and agronomic traits [11]. Several leaf rust resistance genes (*Lr21* (1D), *Lr32* (3D), *Lr39* (2D)) have been transferred into bread wheat from *Ae. tauschii*, including *Lr42* [12]. *Lr42* was introgressed into wheat through a direct cross with *Ae. tauschii* (accession TA 2450) and released as KS91WGRC11 (Century*/TA2450) for further utilization in hexaploid wheat breeding. KS91WGRC11 (carrying *Lr42*) has been successfully used by breeders in several breeding programs [13,14]. Studying near-isogenic lines (NILs) for the *Lr42*, Martin et al. [15] reported that *Lr42* plays an important role in increasing yield, test weight, and kernel weight in wheat. *Lr42* still is one of the highly effective genes, conferring resistance at both seedling and adult plant stages and being used in CIMMYT lines for breeding against leaf rust.

Cox et al. [16] first reported *Lr42* on chromosome 1DS using monosomic analysis in wheat, and it was found to be closely linked to *Lr21*. In their study, Cox et al. [16] reported *Lr42* to be a partially dominant race-specific gene. However, Czembor et al. [17] used Diversity Arrays Technology (DArT) markers to map *Lr42* gene on chromosome 3D and reported that *Lr42* behaved as a dominant gene. By analyzing a set of near-isogenic lines (NILs), Sun et al. [18] mapped *Lr42* on the distal end of chromosome arm 1DS by employing simple sequence repeat (SSR) markers. Using a segregating population of NILs (for *Lr42*) and evaluating for rust infection at both seedling and adult plant stages, they identified three molecular markers—*WMC432*, *CFD15*, and *GDM33*—closely linked to *Lr42*. *WMC432*, about 0.8 cM from *Lr42*, was found to be the closest marker; however, no flanking markers were reported. Further, Liu et al. [19] analyzed an F_2_ population derived from a cross of KS93U50 (*Lr42*)/Morocco to map *Lr42* on chromosome 1DS using six SSR loci with the quantitative calculation method and reported *Lr42* as a recessive gene. *Lr42* was flanked by *WMC432* and *GDM33* onto a 17 cM region at the distil end of chromosome 1DS with the closest proximal marker (*WMC432*) around 4 cM away. Although the previous studies were able to map *Lr42* to wheat chromosome 1DS, the gene lies in a very gene-rich and recombination-hotspot region at the terminal tip of wheat chromosome 1DS; therefore, SSR markers flanking 17 cM regions are not suitable for marker-assisted selection.

The objectives of this study were to (1) determine the genetic and physical location of *Lr42* on the chromosome 1D and (2) develop kompetitive allele-specific polymorphism (KASP) markers to facilitate marker-assisted selection of *Lr42* in wheat breeding programs. This work will lay the foundation for further cloning of the gene.

## 2. Results

### 2.1. Genetic Analysis of Lr42 in Ae. tauschii

The parental lines TA2433 and TA10132 (AL8/78) showed a highly susceptible response to the leaf rust isolate PNMRJ, with an infection type (IT) score of 3, whereas the *Lr42*-carrying accession TA2450 showed highly resistant response with an IT score. Of the 66 F_2_ plants screened from the TA2450/TA2433 population, 50 were resistant and 16 were susceptible, fitting a 3:1 ratio (χ^2^ = 0.20, *p* = 0.89) for a single dominant gene (*Lr42*) segregation in this population (Table 1). Further, the 100 F_2:3_ families also exhibited a good fit for the expected 1:2:1 (resistant:segregating:susceptible) ratio (χ^2^ = 1.92, *p* = 0.38).

The second F_2_ population derived from the cross TA2450/TA10132 showed 53 resistant and 14 susceptible individuals, fitting a 3:1 ratio (χ^2^ = 0.60, *p* = 0.44) and confirming that *Lr42* behaves as a dominant gene (Table 1). Similarly, 100 F_2:3_ families (TA2450/TA10132) evaluated also fit the 1:2:1 (resistant:segregating:susceptible) ratio (χ^2^ = 7.58, *p* = 0.02), with skewing toward resistant families. Our results from segregation of resistance and susceptibility in these two populations suggest that *Lr42* shows dominant inheritance in *Ae. tauschii* backgrounds.

### 2.2. Phenotypic Evaluation of KS93U50/Morocco RIL Population

The hexaploid wheat line KS93U50 carrying *Lr42* showed resistance reaction against isolate PNMRJ producing an infection type (IT) score of 2, whereas Morocco, the susceptible parent of the RIL population, exhibited an IT score of 3+ as expected. The individual plants of 234 F_5_ RILs from the KS93U50/Morocco population were evaluated for responses to PNMRJ and 99 RILs were found to be rust-susceptible, while the other 135 RILs showed a resistant response. The 1S:1R segregation ratio (χ^2^ = 5.54) suggests the presence of a single resistance gene *Lr42* in the RIL population (Table 1).

### 2.3. Marker Discovery and Molecular Mapping

Numerous genomic resources were employed to develop new markers to saturate the target *Lr42* region. The flanking markers for *Lr42*, namely *GDM33*, *WMC432*, and *CFD15* [19], were amplified from Chinese Spring (CS) chromosomes 1D, 4D, and 6D bacterial artificial chromosome (BAC) pools. These pools were developed from a BAC library contructed from fraction-I chromosomes (1D, 4D, and 6D) obtained through flow cytometry separation of CS DNA. *CFD15* was physically mapped to two BAC clones (146DhC878D17 and 146DhC799I02), whereas *WMC432* was mapped to four BAC clones (146DhB488K16, 146DhC808O03, 146DhB488K16, and 146DhB458C07). Both these markers were mapped to the single BAC contig ctg1768; however, the distal marker *GWM33* could not be mapped to a unique BAC contig. Our BAC-based physical map of CS chromosome 1D is anchored to *Ae. tauschii* 10K Infinium SNP-based genetic maps, and we identified BAC contigs proximal and distal to ctg1768 in a 1D physical map (https://urgi.versailles.inra.fr/gb2/gbrowse/wheat_phys_1D_v1/). Six BAC contigs were identified spanning this very terminal region of chromosome 1DS. The 24 BACs in these six contigs were end-sequenced to identify five new SSR markers.

**Figure 1:**
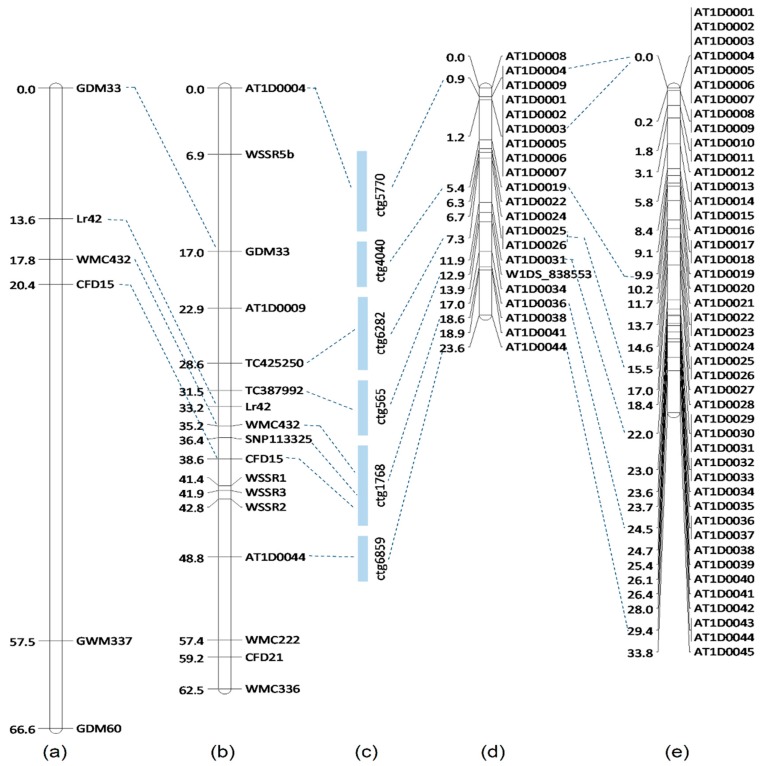
Comparative genetic and physical map of the leaf rust resistance gene *Lr42*: (**a**) genetic map in a KS93U50/Morocco F_2_ population [21]; (**b**) genetic map in a KS93U50/Morocco F_5_ recombinant inbred line (RIL) population (current study); (**c**) physical mapping of the *Lr42* region on Chinese Spring 1D bacterial artificial chromosome (BAC) contigs (current study), and (**d**) *Prelude* (TA2988)/synthetic wheat (TA8051) RIL population (current study), and (**e**) an *Ae. tauschii* AL8/78 (TA10132)/AS75 F_2_ population [19].

We further employed comparative genomic analysis with chromosome 1H of barley [20] for the development of new molecular markers. The collinear interval on chromosome 1H was determined, and 24 genes were predicted to carry plant defense-related domains. Wheat expressed sequence tag (EST) sequences collinear to barley genes were used to develop 19 new EST markers for saturation of the *Lr42* region. In addition, we identified 44 SNPs from *Ae. tauschii* 10K Infinium SNPs mapped on the terminal end of chromosome 1D in a *Prelude* (TA2988)/synthetic wheat (TA8051) RIL population (Figure 1) and also in an *Ae. tauschii* AL8/78 (TA10132)/AS75 F_2_ population [21]. These SNPs were anchored on a CS 1D physical map. Thus, a total of five SSR markers, 19 EST markers, and 44 KASP markers were designed to enrich the candidate region. Of the 68 markers, 11 were polymorphic between KS93U50 and Morocco and used for mapping of *Lr42* (Figure 1, Table 2). We were able to narrow *Lr42* to a 3.7 cM interval flanked by markers *TC387992* and *WMC432* as against the previously reported 17 cM interval on the terminal end of the short arm of chromosome 1D in wheat (Figure 1).

**Figure 2:**
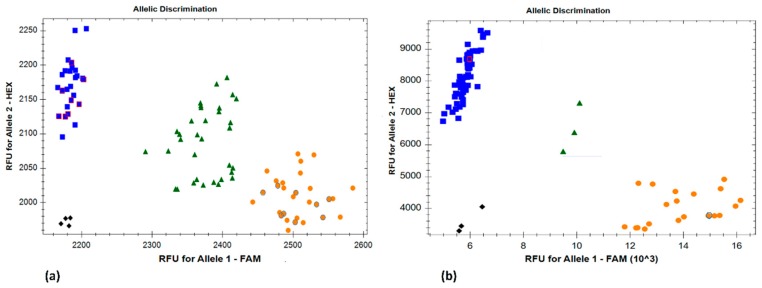
Kompetitive allele-specific polymorphism (KASP) marker *SNP113325* identifying the resistant and susceptible parents and selected progenies of (**a**) an *Ae. tauschii* (TA2450/TA2433) F_2_ population; (**b**) a hexaploid wheat (KS93U50/Morocco) F_5_ RIL population. Blue: susceptible homozygotes; green: heterozygotes; orange: resistant homozygotes; black: non-template controls. The parental resistant (orange with a blue border) and susceptible (blue with a red border) lines have been highlighted in the respective figures.

Further, an additional KASP marker, SNP113325, developed from comparative analysis with barley, was mapped 3.2 cM proximal to *Lr42*, but physically mapped to the same BAC contig as WMC432 and CFD15. The two KASP markers, SNP113325 and TC387992, could be very useful in marker-assisted selection for *Lr42* (Figure 2).

### 2.4. Candidate Genes in the Lr42 Region in Wheat and Ae. tauschii

**Figure 3:**
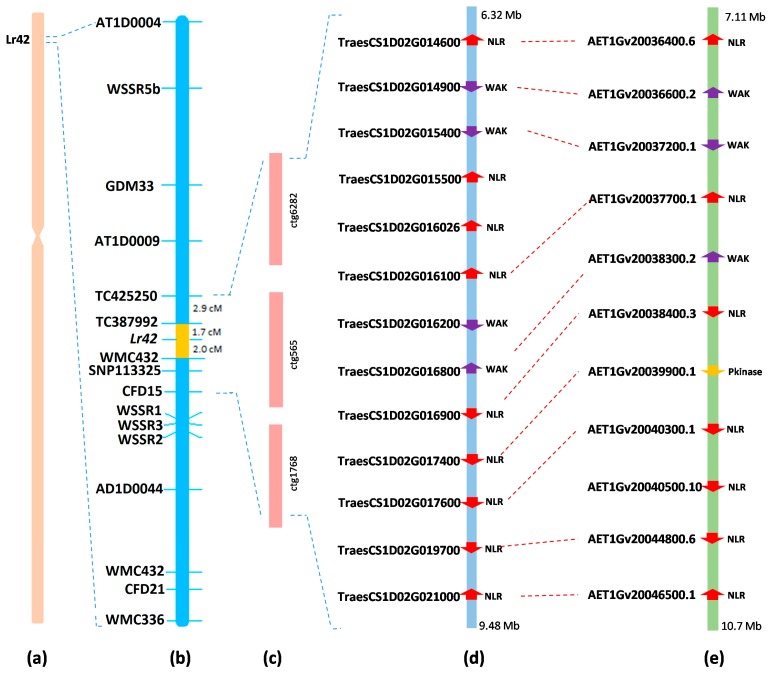
Candidate genes in the *Lr42* region in wheat and *Ae. tauschii*. (**a**) Physical location of *Lr42* on chromosome 1DS of wheat; (**b**) genetic location of *Lr42* in KS93U50/Morocco F5 RIL; (**c**) BAC contigs spanning the *Lr42* physical region; (**d**) annotated genes in the *Lr42* region in CS RefSeq v1.1 [22]; (**e**) annotated genes in the *Lr42* region in the *Ae. tauschii* chromosome 1 sequence [21]. NLR: nucleotide-binding leucine-rich repeats; WAK: wall-associated kinase; Pkinase: protein kinase.

The flanking markers (*TC42520* and *CFD15*) and BAC end sequences from the region flanking *Lr42* were BLASTN searched against CS Wheat RefSeq v1.1 [22]. We identified a corresponding physical segment of 3.16 Mb (6,327,249 bp to 9,490,443 bp) on the tip of the short arm of chromosome 1D of CS Wheat. On the other hand, we identified a 3.5 Mb syntenic *Lr42* region in *Ae. tauschii* chromosome 1. There are 109 high-confidence genes in the 3.16 Mb *Lr42* region based on CS Wheat RefSeq v1.1, of which 23 genes were associated with disease resistance function and three were annotated as serine/threonine protein kinase genes (Table S1). Among the 23 disease resistance genes, 19 genes had NLR domains (associated with most of the rust resistance genes cloned in wheat to date) and another four genes had wall-associated kinase (WAK) domains. Further analysis of these 19 NLR genes showed that 10 genes carry pseudo-NLRs (Table S2); therefore, only nine carry functional NLR domains in the *Lr42* region (Figure 3).

In *Ae. tauschii,* we identified 98 genes in the 3.5 Mb *Lr42* region, with 21 genes encoding plant disease defense-related proteins (Table S3), using the PFAM database [23]. Among the 21 genes, three have putative wall-associated kinase (WAK) domains, seven genes had serine/threonine protein kinase (Pkinase) domains, and 11 genes had NB-ARC domains (Figure 3). Comparative analysis of wheat and *Ae. tauschii* genes in the *Lr42* region showed that four of the 11 NLR genes in *Ae. tauschii* are orthologues of pseudo-NLRs in wheat. Furthermore, the comparison of genes from two species showed that six NLRs and two WAKs from wheat have high sequence similarity (>96%) and are orthologues in *Ae. tauschii*. One NLR gene from wheat could be an orthologue of a protein kinase (Pkinase) gene from *Ae. tauschii* as it shared a sequence similarity of 90%. Apart from these genes, no orthologues were found in *Ae. tauschii* for one WAK and two NLR genes that were present in wheat. By contrast, one NLR gene was present in *Ae. tauschii* but absent in wheat (Figure 3).

## 3. Discussion

Leaf rust can cause severe losses in wheat yield and grain quality. Host resistance is a key component in managing leaf rust, and thus, molecular genetic characterization of resistance and identification of tightly linked molecular markers can help achieve better understanding of the mechanism of leaf rust resistance and facilitate marker-assisted breeding and gene pyramiding. Liu et al. [19] mapped *Lr42* on the short arm of wheat chromosome 1D after analyzing F_2_ and F_3_ generations of KS93U50/Morocco population. In the current study, we advanced the population to F_5_ RILs. Segregation observed in F_2_ and F_3_ generations of the KS93U50/Morocco population as studied by Liu et al. [19] suggested that *Lr42* was recessive. In the current study, we evaluated two independent *Ae. tauschii* F_2_ populations (TA2450/TA2433 and TA2450/TA10132) for a response to leaf rust using the same isolate, PNMRJ. In both the populations, the majority of plants were resistant, indicating a dominant monogenic control of the resistance reaction. Further segregation pattern in the F_2:3_ generation in the two *Ae. tauschii* populations was consistent with F_2_, suggesting that *Lr42* behaves as a dominant gene in *Ae. tauschii*. *Ae. tauschii* (the D-genome donor of wheat) is the source of *Lr42*, however, the segregation behavior of *Lr42* had not been studied earlier in *Ae. tauschii*. There are several conflicting reports regarding the segregation behavior of the *Lr42* gene in hexaploid wheat. Cox et al. [16] reported that *Lr42* was partially dominant, whereas Czembor et al. [17] reported it as being a dominant gene in wheat. However, these studies used several different genetic backgrounds or different leaf rust isolates, which could have resulted in the differences, as demonstrated by Kolmer and Dyck [24].

Applying BAC-based physical mapping, BAC-end sequencing, and comparative genomic analysis with barley [20], we identified 68 new markers and further mapped *Lr42* to a 3.7 cM interval. Liu et al. [19] reported *Lr42* as being located on the distal tip of chromosome 1DS flanked by a 17 cM interval. In the current study, we significantly reduced the size of the *Lr42* flanking segment by mapping *TC387792* and *WMC432,* as being 1.7 cM and 2.0 cM away from *Lr42* on the distal and proximal regions, respectively. Additionally, *SNP113325* lies 3.2 cM distal to *Lr42* and is also tightly linked to *Lr42* in the RIL population. The two KASP markers *SNP113325* and *TC387792* have been used for screening of hard winter wheat in regional nurseries (see 2018 SRPN, Table 10 from https://www.ars.usda.gov/ARSUserFiles/30421000/HardWinterWheatRegionalNurseryProgram/2018%20SRPN%20021519.xlsx) in marker-assisted selection for *Lr42* in wheat breeding programs.

We further physically delimited the *Lr42* region to a 3.16 Mb (6,327,249 bp to 9,490,443 bp) region in chromosome 1D using the Chinese Spring reference genome RefSeq v1.1 [22]. As expected, *Lr42* is present in a high-recombination region with a higher genetic-to-physical map ratio, making the search for the candidate gene relatively easy compared to the centromeric region. Though we identified 109 genes in this region, 23 of these genes have kinase domains mostly associated with disease-related genes in wheat. Besides the 23 genes, three genes were annotated as protein kinases (Pkinases); however, Pkinase genes have not been associated with wheat rust resistance genes to date. Out of the 23 genes, 10 genes were found to be pseudo-NLRs, leaving only 13 candidate genes. Of the final 13 candidate genes, nine genes have NLR domains and four genes have WAK domains. In *Ae. tauschii*, we identified seven genes with NLR domains that could be candidates for *Lr42*. Currently, several rust resistance genes have been cloned in wheat, including *Lr10* [25], *Lr21* [26], *Lr1* [27], *Lr22a* [28], *Sr33* [29], *Sr35* [30], *Sr45* [31], and *Yr10* [32], and all encode an NLR-type protein. Though wall-associated kinase domains (WAKs) have been reported to confer resistance to fungi in wheat, such as *Snn1* against Septoria nodorum blotch [33] and *Stb6* against *Septoria tritici* blotch [34], none of them showed resistance to wheat rusts. Therefore, the nine NLR-type genes in the *Lr42* region are the most probable candidates for the *Lr42* gene. Nonetheless, it is possible that *Lr42* has a regulatory mechanism different from NBS-LRR type genes. Molecular cloning of *Lr42* is thus required to reveal the complete regulation mechanism, which can be facilitated using novel cloning techniques such as MutRenSeq [31] or TACCA [28]. These techniques have been recently used to clone several disease-related genes in wheat, such as *Lr22a* [28] and *Pm2* [35]. Thus, we have identified an *Lr42* susceptible mutant by EMS mutagenesis of *Ae. tauschii* accession TA2450, and future efforts will be made to further characterize *Lr42*.

*Lr42* is physically located on the distal region of wheat chromosome 1DS, where a number of disease-related genes have been mapped to, such as *Lr21* [26], *Sr33* [29], *Sr45* [31], *Pm24* [36], and several other genes of agronomic importance. In wheat, the terminal gene-rich regions have been found to be positively correlated with recombination frequency [37]. Identification of tightly linked flanking markers could facilitate marker-assisted selection of *Lr42* in wheat breeding programs. The KASP markers developed in our study closely flank *Lr42* and could be efficiently used for marker-assisted selection of *Lr42* and help in stacking of the *Lr42* gene with other race non-specific disease resistance genes to develop wheat cultivars with more durable resistance against leaf rust.

## 4. Materials and Methods

### 4.1. Plant Materials and Rust Resistance Evaluation

*Lr42* was identified from *Ae. tauschii* accession TA2450 [16]. We screened several *Ae. tauschii* accessions with leaf rust race PNMRJ and identified two *Ae. tauschii* accessions, TA2433, and TA10132 (AL8/78), that were susceptible to PNMRJ (PNMRJ is avirulent to *Lr42*). TA2450 was then crossed to TA2433 and TA10132 to develop two F_2_ populations (TA2450/TA2433 and TA2450/TA10132). A total of 100 F_2_ plants were derived from each cross (TA2450/TA2433 and TA2450/TA10132), of which a sample of around 70 plants were artificially inoculated with leaf rust race PNMRJ in a greenhouse following the method described by Liu et al. [19]. Briefly, the F_2_ plants from each of the two crosses, along with the parental lines, were planted in plastic trays. The plants were artificially inoculated with PNMRJ at the two-leaf stage. For artificial inoculation, the PNMRJ spores were suspended in Soltrol 170 mineral oil (CPChemicals LLC, Garland, TX, USA). The suspension was sprayed on the seedlings using a pressure sprayer, followed by incubation at 20 °C for 24 h in a humid chamber. Following incubation, the F_2_ plants were grown in a greenhouse at 20–24 °C for the establishment of infection. At ten days after inoculation, the seedlings were scored for rust infection type (IT) on a 1 to 4 scale (; = hypersensitive flecks, 1 = small uredinia with necrosis, 2 = moderate size pustules with chlorosis, 3 = moderate-large size uredinia without necrosis or chlorosis, and 4 = large uredinia lacking necrosis or chlorosis) [38,39]. The scoring was repeated after two days for confirmation. For further study, F_2:3_ progenies for each cross were grown and evaluated for leaf rust to identify homozygous non-segregating families. About 20 seeds for each family were grown and evaluated using the same procedure used for the F_2_ populations. Based on the reactions of parental lines to PNMRJ, the plants with IT ≤ 2 were considered resistant and those with IT > 2 susceptible.

In addition to the two *Ae. tauschii* populations, we used the F_5_ RIL population of 234 progenies developed from the wheat cross KS93U50/Morocco. The RIL population, along with parents KS93U50 and Morocco, were grown and evaluated for leaf rust resistance under controlled greenhouse conditions with three replications. The F_5_ RILs were advanced lines from the F_2_ population analyzed by Liu et al. [19] to map *Lr42*. The inoculation and scoring method was the same as described for *Ae. tauschii* populations. The data from the three populations were used to deduce the inheritance pattern of *Lr42*.

### 4.2. Marker Discovery and Saturation in the Candidate Region

Previously reported *Lr42* flanking markers [19] were mapped on to the physical map of chromosome 1D of Chinese Spring wheat (https://urgi.versailles.inra.fr/gb2/gbrowse/wheat_phys_1D_v1/) by identifying the BACs associated with the candidate region using three-dimensional BAC pools. Identified BACs spanning this region were end-sequenced to generate five new PCR-based SSR markers using the DesignPrimer tool (https://kofler.or.at/bioinformatics/SciRoKo/DesignPrimer.html). Further, the genomic information from the BACs and comparative analysis with chromosome 1H of barley [20] was conducted to develop an additional 19 EST markers. In addition, KASP markers were developed from *Ae. tauschii* 10K Infinium single nucleotide polymorphisms (SNPs) [19] that were mapped onto the BACs of this region. A total of 68 new molecular markers, along with previously reported markers, were used to genotype the F_5_ RILs along with the parents. The polymorphic markers were used for the construction of a genetic map for the *Lr42* region. The EST markers that were polymorphic between parents were later converted into KASP-based markers for easy and reliable genotyping and to select *Lr42* in breeding programs.

### 4.3. DNA Extraction and Genotyping of the RIL Population

Ten-day-old uninfected seedlings of RILs, along with parental lines, were used for DNA extraction using the cetyl trimethyl ammonium bromide (CTAB) method [40]. Extracted DNA samples were quantified using a Nanodrop ND1000 spectrophotometer (Nanodrop Technologies, US) for normalization of DNA concentration. For SSR and EST genotyping, polymerase chain reaction (PCR) was carried out in a GeneAmp PCR System 9700 (Applied Biosystems, CA, USA) using 20 µL of reaction mixture containing 100 ng genomic DNA, 25 ng each of forward and reverse primers, and 10 µL of 2× PCR Mastermix. Thermocycling profile was set as follows: initial denaturation at 95 °C for 5 min; 30 cycles each at 95 °C for 30 sec, 50–61 °C (depending upon the annealing temperature of particular primers) for 30 sec, 72 °C for 1 min; and a final extension step at 72 °C for 7 min. The EST and SSR PCR products were visualized using 3% high-resolution agarose gel (GeneMate, ISC Bioexpress, Inc, Kaysville, UT, USA). The kompetitive allele-specific polymorphism (KASP) genotyping was carried out using 8 µL of total reaction mixture containing 3 µL of 20–25 ng/µL genomic DNA, 5 µL of KASP Mastermix (LGC Genomics, Teddington, UK) consisting of a FAM and HEX specific FRET cassette, Taq polymerase and optimized buffer; and 0.07 µL of KASP assay mix consisting of two allele-specific primers and one common primer. For accurate distribution of the small volume of assay mix, KASP Mastermix and assay mix were combined before dispensing. PCR was carried out using a Bio-Rad CFX96 Real-time PCR system (Bio-Rad Laboratories, Hercules, CA, USA). The PCR profile was designed as follows: 94 °C for 20 min (hot start activation); 10 touchdown cycles each at 94 °C for 20 sec, 61–55 °C for 60 sec (dropping by 0.6 °C per cycle); followed by 35 cycles at 94 °C for 20 sec and 55 °C for 60 sec. Bio-Rad CFX Manager (Bio-Rad Laboratories, USA) was used for reading the plates after PCR.

### 4.4. Statistical Analysis and Genetic Mapping

Pearson’s chi-squared analysis was used to test the goodness of fit for observed frequencies to the expected genetic frequencies. The chi-squared analysis was performed in R ver 3.4 [41] using the function ‘chisq.test’. CarthaGene v1.3 was employed for the construction of genetic maps and automatical marker ordering to obtain a multipoint maximum likelihood map [42]. Firstly, a logarithm of odds (LOD) score of 3.0 was used as a threshold value for identifying the linkage groups, followed by ordering of the markers using the Kosambi mapping function [43]. The genetic linkage map was improved using the ‘simulated annealing’ algorithm and ‘verification algorithms’ in Carthagene [42]. MapChart version 2.2 [44] was used to draw and combine different genetic maps.

### 4.5. Physical Mapping of the Lr42 Region on Chromosome 1DS

Using BAC-end sequences and the sequences of EST markers flanking *Lr42*, we identified the *Lr42* region in the hexaploid wheat Chinese Spring (CS) chromosome 1D RefSeq v1.1 [22] and the *Ae. tauschii* reference genome sequence [45] using BLASTN [46]. Gene annotation of the *Lr42* genomic interval on CS chromosome 1D of wheat [22] and *Ae. tauschii* chromosome 1 [45] was obtained to identify candidate disease-resistance genes in the region.

## 5. Conclusions

In the current study, we fine-mapped the leaf rust resistance gene *Lr42* to a 3.7 cM region from the previously reported 17 cM region on chromosome 1DS in wheat. We further physically mapped the *Lr42* region on Chinese Spring RefSeq v1.1 and *Ae. tauschii* reference genomes and identified genes with defense-related functions as possible candidates for *Lr42*. The KASP markers flanking *Lr42* developed in the current study will facilitate marker-assisted selection of *Lr42* and pyramiding of the gene with other adult plant resistance genes for effective management of leaf rust in wheat.

## Figures and Tables

**Table 1:** Segregation of *Lr42* in *Ae. tauschii* and hexaploid wheat populations against leaf rust race PNMRJ. The observed and expected ratios correspond to the resistance:susceptible in F_2_ generations and homozygous resistant:segregating:homozygous susceptible in F_2:3_ generations.

Sr No.	Species	Population	Generation	Lines Evaluated	Observed Ratio	Expected Ratio	χ^2^	*p*-Value*
1	*Ae. tauschii*	TA2450/TA2433	F_2_	66	50:16	3:1	0.20	0.89
			F_2:3_	100	27:54:19	1:2:1	1.92	0.38
2	*Ae. tauschii*	TA2450/TA10132 (AL8/78)	F_2_	67	53:14	3:1	0.60	0.44
			F_2:3_	100	33:53:14	1:2:1	7.58	0.02
3	*T. aestivum*	KS93U50/Morocco	F_5_ RIL	234	99:135	1:1	5.54	0.02

* α = 0.01.

**Table 2:** Simple sequence repeat (SSR) and KASP markers developed and mapped on the KS93U50/Morocco RIL population.

Sr. No.	Primer Name	Assay Type	Sequence(s)
1	WSSR1	SSR	ACGACGTTGTAAAACGACTGGAGACAGACGAACGCATA
			TGCATGCATACACACACCAG
2	WSSR2	SSR	ACGACGTTGTAAAACGACAGCAATGCAGTTGCAAAGAG
			GCAAAGATGGACAGATGGCT
3	WSSR3	SSR	ACGACGTTGTAAAACGACAAGATCAGCTCCGACAGCTC
			CGAAGTCAGCACAAACCAAA
4	WSSR5	SSR	ACGACGTTGTAAAACGACTGGTGAATCTTGCACCACAT
			CTGGACACCGTTCGTTAGGT
5	AT1D004	KASP	GGTACCATGTTGTTTCGCATGTCTAT
			GTACCATGTTGTTTCGCATGTCTAC
			GGAGGCAGAGACAATAAGTTTATGTTACAA
6	AT1D0009	KASP	GGAGATCTTTATATTTGTGGTTTGCCA
			GAGATCTTTATATTTGTGGTTTGCCG
			CCAGGTCACAGGCTGTGATGTTTAA
7	TC425250	KASP	GCACTACTTTTATTGATGTTGTGTAACC
			AAGCACTACTTTTATTGATGTTGTGTAACT
			CAGAGGGAAGAAAACAACACTGAACAAAA
8	TC387992	KASP	TTGGATCTGCATTCCTTCTCCCA
			GGATCTGCATTCCTTCTCCCG
			CTTTGGGATGTTGCTGCTGGAGAT
9	SNP113325	KASP	GGTGTTTGGCAGCATCATCACG
			GGTGTTTGGCAGCATCATCACC
			GACAACTTGAGACACTAGATATCAGAGAT

## Supplementary Materials

Supplementary materials can be found at https://www.mdpi.com/1422-0067/20/10/2445/s1.

## Abbreviations

MAS	Marker-assisted selection
KASP	Kompetitive allele-specific PCR
BAC	Bacterial artificial chromosome
EST	Expressed sequence tag
SSR	Simple sequence repeat
